# The Predictive Role of *PIK3CA* Mutation Status on *PI3K* Inhibitors in *HR+* Breast Cancer Therapy: A Systematic Review and Meta-Analysis

**DOI:** 10.1155/2020/1598037

**Published:** 2020-05-10

**Authors:** Mingming Wang, Jin Li, Jiangsheng Huang, Mei Luo

**Affiliations:** ^1^Department of Minimally Invasive Surgery, The Second Xiangya Hospital, Central South University, Changsha, Hunan, China; ^2^Department of Anesthesiology, The Third Xiangya Hospital, Central South University, Changsha, Hunan, China; ^3^Department of Neurosurgery, Xiangya Hospital, Central South University, Changsha, Hunan, China

## Abstract

**Aim:**

To evaluate the impact of *PIK3CA* mutation status on clinical outcomes of *HR+* breast cancer treated with *PI3K* inhibitors.

**Methods:**

A comprehensive literature search was conducted in online databases from inception to December 31, 2019. The main characteristics and prognostic data of each eligible study were extracted. The odds ratio (OR) for the overall response rate (ORR) and hazard ratio (HR) for progression-free survival (PFS) were estimated using the fixed-effects Mantel-Haenszel model.

**Results:**

A total of 8 studies involving 2670 patients were included for analysis. Overall, the clinical outcomes of *PI3K* inhibitors were significantly influenced by *PIK3CA* mutation status in *HR+* breast cancer. After the treatment of *PI3K* inhibitors, *HR+* breast cancer patients with *PIK3CA* mutations presented better ORR (*PIK3CA-*mutated group: OR = 1.98 [95% CI, 1.46 to 2.70]; *PIK3CA* wild-type group: OR = 1.09 [95% CI, 0.78 to 1.53]) and better PFS (*PIK3CA*-mutated group: HR = 0.65 [95% CI, 0.55 to 0.76]; *PIK3CA* wild-type group: HR = 0.87 [95% CI, 0.70 to 1.09]). No publication bias was detected for ORR and PFS in our analysis.

**Conclusion:**

In this meta-analysis, it suggests that the association between clinical outcomes of *PI3K* inhibitors and *PIK3CA* mutation status is dramatic. *PIK3CA* mutations were a favorable factor in the clinical outcomes of *HR+* breast cancer treated with *PI3K* inhibitors.

## 1. Introduction

Breast cancer is the most commonly diagnosed cancer in women worldwide. More than 80% of breast cancer is classified as hormone receptor-positive (*HR+*) breast cancer [1]. Current therapeutic strategies for *HR+* breast cancer comprise endocrine therapy, *mTOR*, or cyclin-dependent kinase 4/6 (*CDK4/6*) inhibition et al. Nevertheless, disease progression, metastasis, and drug resistance eventually develop [2]. To our knowledge, diversified pathways (e.g., *RAS/MAPK*, *NFκB*, or *PI3K/AKT/mTOR*) get involved in drug resistance to current therapy [3]. Monotherapy targeting a single pathway can be easily failed to generate clinical benefit due to the aberrant activation of bypass signaling. In particular, *PI3K/AKT/mTOR* is the most frequently altered pathway in *HR+* breast cancer [4]. Therefore, inhibition of the *PI3K/AKT/mTOR* signaling pathway should be of great priority to overcome therapeutic challenges in *HR+* breast cancer [5].

Previous clinical data have elucidated the clinical efficacy of the *PI3K/AKT/mTOR* pathway inhibition in *HR+* breast cancer therapy. For example, administration of everolimus (a rapamycin analog inhibitor of *mTOR*) can increase the efficacy of letrozole in the endocrine-resistant form [6]. A combination of everolimus with tamoxifen can improve the survival outcome for *OR+* metastatic breast cancer patients previously treated with aromatase inhibitors [7]. And the addition of everolimus to exemestane can also improve the survival outcome for breast cancer patients [8]. Besides the *mTOR* inhibition, oral inhibitors for *PI3K* isoforms have been established for the treatment of *HR+* breast cancer. Those inhibitors specifically bind the phosphatidylinositol (3,4,5)-trisphosphate (*PIP3*), which mediates the downstream signaling of *PI3K*. It has shown synergistic antitumor activity with endocrine therapy against *HR+* breast cancer in both preclinical and clinical results [9]. Plenty of gene mutations, such as *PIK3CA* mutations, often occurred in *HR+* breast cancer and associated with the clinical efficacy of therapeutic strategies. Based on the results of current research, the *PI3K/AKT/mTOR* pathway can be activated through *PIK3CA* mutations [10]. Some studies report that *PIK3CA* mutations can inactivate the downstream components, such as *TORC1*, which can generate a better outcome in endocrine-resistant breast cancer [11]. Nevertheless, the BOLERO-2 study has debated that there is no association between *PIK3CA* mutation status and clinical benefit of mTOR inhibition. Similarly, the predictive role of *PIK3CA* mutation status on the clinical efficacy of *PI3K* inhibitors remains controversial in *HR+* breast cancer therapy from current results [12].

According to preclinical and clinical results, whether *PIK3CA* mutation status can be a predictive role for *PI3K* inhibitors remains debating. Herein, identification of the predictive role of *PIK3CA* mutation status on the clinical outcomes of *PI3K* inhibitors should be of great priority to benefit the diagnosis and prognosis for patients with *HR+* breast cancer.

## 2. Materials and Methods

### 2.1. Literature Search Strategy

This meta-analysis was conducted according to the Preferred Reporting Item for Systematic Reviews and Meta-Analyses (PRISMA) statement. We followed the methods of Zou et al. [13]. All reviewed articles were retrieved from PubMed, Cochrane, and Web of Science. Privately and publicly funded clinical studies recorded in *ClinicalTrials.gov* were also screened. Following terms were applied to comprehensively seize the articles: (1) (Breast Neoplasm) or (Neoplasm, Breast) or (Breast Tumors) or (Breast Tumor) or (Tumor, Breast) or (Tumors, Breast) or (Neoplasms, Breast) or (Breast Cancer) or (Cancer, Breast) or (Mammary Cancer) or (Cancer, Mammary) or (Cancers, Mammary) or (Mammary Cancers) or (Malignant Neoplasm of Breast) or (Breast Malignant Neoplasm) or (Breast Malignant Neoplasms) or (Malignant Tumor of Breast) or (Breast Malignant Tumor) or (Breast Malignant Tumors) or (Cancer of Breast) or (Cancer of the Breast) or (Mammary Carcinoma, Human) or (Carcinoma, Human Mammary) or (Carcinomas, Human Mammary) or (Human Mammary Carcinomas) or (Mammary Carcinomas, Human) or (Human Mammary Carcinoma) or (Mammary Neoplasms, Human) or (Human Mammary Neoplasm) or (Human Mammary Neoplasms) or (Neoplasm, Human Mammary) or (Neoplasms, Human Mammary) or (Mammary Neoplasm, Human) or (Breast Carcinoma) or (Breast Carcinomas) or (Carcinoma, Breast) or (Carcinomas, Breast); and (2) (*PIK3CA*) or (Phosphoinositide-3-kinase catalytic alpha polypeptide) or (Phosphoinositide-3-kinase catalytic alpha polypeptide). References of included studies were conditionally screened. On-topic articles were included while off-topic items were removed.

### 2.2. Inclusion Criteria

Eligible studies were (1) patients who were diagnosed with *HR+* breast cancer, (2) patients who were treated with PI3K inhibitors, (3) the *PIK3CA* mutation status was detected, and (4) data about the overall response rate (ORR) and progression-free survival (PFS) in *PIK3CA*-mutated group and *PIK3CA* wild-type group were reported accordingly. Our exclusion criteria were (1) review, letters, comments, conference abstracts, or articles without outcomes of interest and (2) duplicate or overlapping data. If several publications from the same project were identified simultaneously, the newest version and the most comprehensive data would be included.

### 2.3. Data Extraction and Quality Assessment

Data were independently extracted by two reviewers (Mingming Wang and Jin Li) from the included studies. The following items were extracted from the text and supplementary materials: first author's name, publication year, study type, *PI3K* inhibitor regimen, and specific clinical outcomes. The Newcastle-Ottawa Scale (NOS) was employed to assess the quality of the included studies. Each study was reckoned according to selection, comparability, and outcome. Any discrepancies were solved by mutual discussion.

### 2.4. Statistical Analysis

The primary outcomes of interest were ORR and PFS for *HR+* breast cancer patients in the *PIK3CA* wild-type group and *PIK3CA*-mutated group. RevMan 5 (Revman, Cochrane Collaboration; Oxford, England) was applied to perform statistical analysis. The pooled odds ratio of ORR and hazard ratio of PFS were presented to evaluate the difference between the *PIK3CA*-mutated group and the *PIK3CA* wild-type group. Heterogenicity between studies was assessed by the Chi-squared test and *I*^2^. A fixed-effects model was applied when there was no significant heterogenicity (*I*^2^ < 50% or *p* value > 0.05). Otherwise, the random-effects model was in use.

## 3. Results

### 3.1. Literature Search and Study Selection

**Figure 1:**
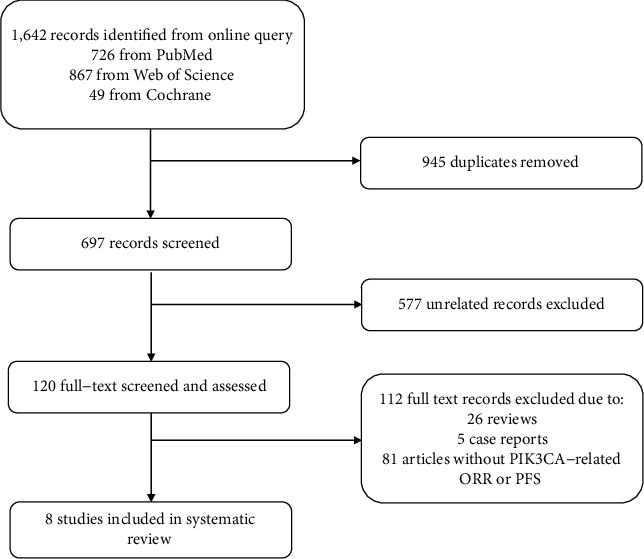
Online search strategy.

The primary search strategy identified 1,642 records after an online inquiry. After removing 945 duplicates and excluding 577 irrelevant records, we included 120 articles after review in the title and abstract. The full text of the remaining 120 articles was meticulously screened and assessed. 112 pieces of literature were excluded due to the following reasons: reviews, case reports, conference abstracts, or without outcomes of interest. Ultimately, 8 studies were selected according to the inclusion and exclusion criteria. Details for the online search strategy were exhibited in Figure 1.

### 3.2. Main Characteristics of the Included Studies

The main characteristics of the 8 included studies were summarized in Table 1. All included studies were published before December 31, 2019. All patients were diagnosed as *HR+* breast cancer. The study type of each study and the available register number for each clinical trial were presented. The detailed *PI3K* inhibitor regimen is shown in our table. Six studies were applied to evaluate the survival outcome, and 7 studies were involved to assess the responsive outcome. According to the score, 3 studies had quality scores of 7 or higher while others were less than 7.

### 3.3. Relationship between *PIK3CA* Mutations and Overall Response Rate (ORR)

**Figure 2:**
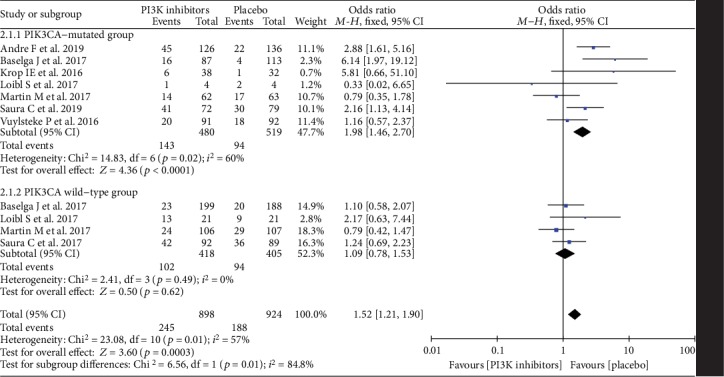
Odds ratio for overall response rate (ORR) in the *PIK3CA*-mutated group and the *PIK3CA* wild-type group.

A total of 7 studies reported data about the overall response rate (ORR) of *PI3K* inhibitors in the *PIK3CA-*mutated group and/or *PIK3CA* wild-type group. Pooled ORR demonstrated that *PI3K* inhibitors generated 1.98 [95% CI, 1.46 to 2.70] odds ratio (OR) in *HR+/PIK3CA*-mutated breast cancer patients and 1.09 [95% CI, 0.78 to 1.53] odds ratio (OR) in *HR+/PIK3CA* wild-type breast cancer patients. Medium heterogeneity (*p* = 0.02, *I*^2^ = 60%) presented in the *PIK3CA*-mutated group, while no heterogeneity was observed in the *PIK3CA* wild-type group (*p* = 0.49, *I*^2^ = 0%). The ORR of *PI3K* inhibitors between the *PIK3CA*-mutated group and the *PIK3CA* wild-type group was statistically different (*p* = 0.01, *I*^2^ = 84.8%) in Figure 2.

### 3.4. Relationship between *PIK3CA* Mutations and Progression-Free Survival (PFS)

**Figure 3:**
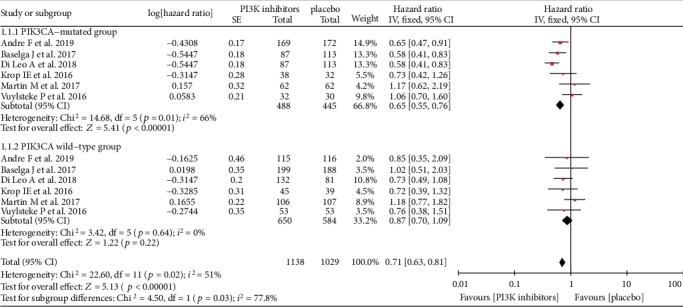
Hazard ratio for progression-free survival (PFS) in the *PIK3CA*-mutated group and the *PIK3CA* wild-type group.

A total of 6 studies provided data about the progression-free survival (PFS) of *PI3K* inhibitors in HR+ breast cancer according to *PIK3CA* mutation status. Comparing with the hazard ratio (HR) of PFS in the *HR+/PIK3CA* wild-type group (HR = 0.87 [95% CI, 0.70 to 1.09]), the PFS of *PI3K* inhibitors was further improved in the *HR+/PIK3CA*-mutated group (HR = 0.65 [95% CI, 0.55 to 0.76]). Medium heterogeneity (*p* = 0.01, *I*^2^ = 66%) existed in the *PIK3CA*-mutated group, while no heterogeneity was observed in the *PIK3CA* wild-type group (*p* = 0.64, *I*^2^ = 0%). The improvement of PFS by *PI3K* inhibitors in the *PIK3CA*-mutated group was significantly better than the *PIK3CA* wild-type group (*p* = 0.03, *I*^2^ = 77.8%) in Figure 3.

### 3.5. Publication Bias

**Figure 4:**
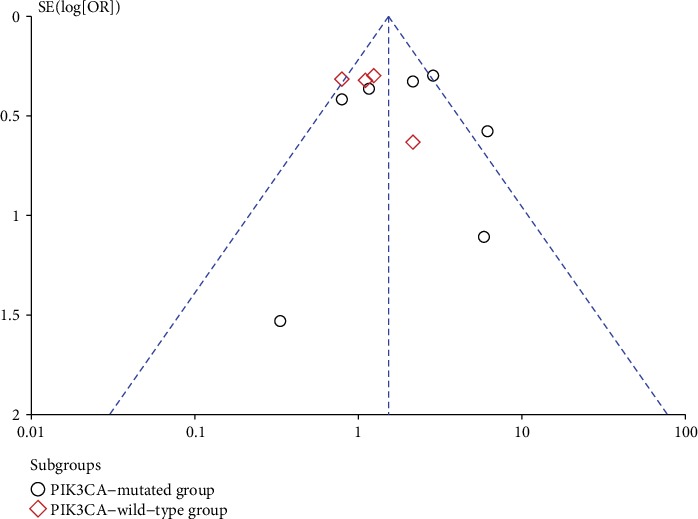
Funnel plot for overall response rate (ORR) analysis.

**Figure 5:**
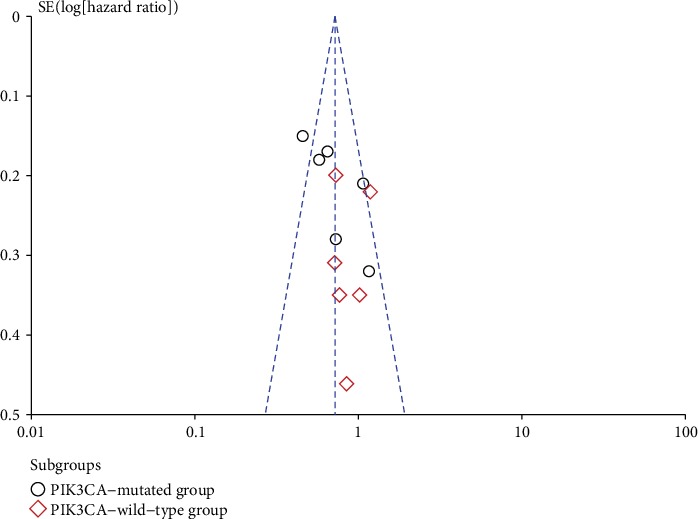
Funnel plot for progression-free survival (PFS) analysis.

Funnel plots were applied to assess whether publication bias existed in our analysis. The funnel plots did not indicate any publication bias for ORR in Figure 4 and PFS in Figure 5. The visual estimation of the funnel plots showed clear symmetry.

## 4. Discussion

As the most exhaustive meta-analysis to investigate the efficacy of *PI3K* inhibitors in *HR+* breast cancer according to *PIK3CA* mutation status, our results demonstrate that the responsive and survival outcomes of *PI3K* inhibitors can be significantly improved in *PIK3CA*-mutated population comparing with wild-type *PIK3CA* population. Based on our findings, *PIK3CA* mutation status can be a prognostic factor for *HR+* breast cancer patients treated with *PI3K* inhibitors. However, further studies are still required to confirm our findings.

To our knowledge, multiple signaling pathways are implicated in malignant progression and drug resistance in breast cancer. Inhibition of those aberrantly activated pathways is an important therapeutic strategy to manage breast cancer. For example, blockade of the hyperactivated *PI3K/AKT/mTOR* pathway by diversified inhibitors should bring up some clinical benefits for breast cancer patients. In 2012, *mTOR* inhibitors (everolimus) showed a profitable effect on endocrine therapy of breast cancer therapy [14]. However, preliminary data on *mTOR* inhibitors illustrate that *mTOR* inhibition elicits *AKT* phosphorylation via feedback activation. *AKT* phosphorylation often induces drug resistance of *mTOR* inhibitors. The importance of developing novel functional biomarkers to enhance therapeutic benefit has been highlighted. Treatment with direct *PI3K* inhibitors could attenuate or abrogate *AKT* phosphorylation, which might potentially vanquish the resistance to mTOR inhibitors [15].

Latterly, plenty of *PI3K* inhibitors have been established to treat *HR+* breast cancer. Current *PI3K* inhibitors are mainly categorized into 2 types: one is pan-*PI3K* inhibitors, which targets all four isoforms (p110*α*, *β*, *γ*, and *δ*) of *PI3K* (e.g., pictilisib and buparlisib). Another is selective-*PI3K* inhibitors, which selectively recognize the mutated *PI3Kα* subunit and wild-type *PI3K* (e.g., alpelisib and taselisib) [16, 17]. The specificity, binding affinity, and efficacy of each inhibitor are varied. According to current reports, pan-*PI3K* inhibitors often result in dose reduction and discontinuation due to the high rate of severe adverse events [18, 19]. Rationally, those limitations can be renovated by selective-*PI3K* inhibitors [20]. A better safety profile permits prolonged administration at higher doses and subsequently advances clinical efficacy [21]. For example, alpelisib is an equipotent selective inhibitor against both the wild-type and mutated *PI3Kα* subunit [22]. And taselisib displays greater selectivity for mutated than wild-type *PI3Kα* and a less potent inhibition to *PI3Kβ* isoform [23]. Those selective inhibitors show better clinical benefit not only as a single agent but also in combination with fulvestrant or letrozole. Since the negative clinical results about pan-*PI3K* inhibitors have prevented further investigation, more clinical studies about selective-*PI3K* inhibitors may support our findings on the predictive role of *PIK3CA* mutation status in breast cancer patients [24].

A growing number of clinical studies support that *PIK3CA* mutation favors the responsive and survival outcome in *PI3K* inhibitor treatment [25], while some studies reported that the clinical efficacy of *PI3K* inhibitors is not affected by the *PIK3CA* mutation status [26]. The ability of *PIK3CA* mutation status to predict the benefit of *PI3K* inhibitors is imperative for further subclassification of *HR+* breast cancer. A panel of *PIK3CA* mutations in exons 1, 7, 9, and 20 have been detected and analyzed via current technology (e.g., R88Q in exon 1, N345K in exon 4, C420R in exon 7, E542K in exon 9, E545K/A/G/D in exon 9, Q546K/E/R/L in exon 9, M1043I in exon 20, H1047R/L/Y in exon 20, and H1049R in exon 20) [22]. Notably, the limitation of different strategies to confirm the *PIK3CA* mutation status should be addressed. Since archived tumors are unlikely to present the real-time *PIK3CA* mutation status, fresh biopsies are more representative of mutation status [27]. However, additional surgery is not often applicable or desirable in patients with advanced disease or metastasis. Therefore, circulating tumor DNA (ctDNA) analysis has been widely employed as a noninvasive approach to detect *PIK3CA* mutation status [28]. Comparing with archival tumor samples, ctDNA analysis is a more illustrative and less invasive technique to assess the mutational status and heterogenicity throughout the treatment [29]. In our methods, we collect the responsive and survival data according to the *PIK3CA* mutation status in ctDNA analysis, which is broadly applied to detect the gene mutation status in cancers.

We have well-demonstrated the favorable role of *PIK3CA* mutations for *PI3K* inhibitors in *HR+* breast cancer therapy, whereas our study still has some limitations that should be concerned. Firstly, the disease context, concomitant therapy, and the specificity of each inhibitor should be of great interest in establishing the predictive value of *PIK3CA* mutation status. Those factors were not indexed by subgroup analysis due to a lack of efficient data. In the future, all of those affective factors should be taken into consideration and well classified. Secondly, the aberrant activation of the *PI3K/AKT/mTOR* pathway often occurs via various mechanisms, such as the alteration of *PI3K* catalytic subunits (*PIK3CA* and *PIK3CB*), *AKT1*, *AKT2*, or *PTEN*. The crosstalk between *PIK3CA* mutations with other gene alterations is not discussed in our analysis. Regardless of alterations in the *PI3K/AKT/mTOR* pathway, a further determination of underlying gene alterations should be of obligation to develop the novel therapeutic strategy in breast cancer [30]. The definition of the subsets of breast cancer according to their gene alteration might be essential to maximize the clinical benefit for those patients. Finally, we only assess the responsive and survival outcomes of *PI3K* inhibitors in *HR+* breast cancer. However, the biological outcomes are surrogate endpoints strongly linked with responsive and survival outcomes [31]. In future studies, the evaluation of those biological outcomes should be an essential supplement to draw a more solid conclusion in this area.

## 5. Conclusion

The *PIK3CA* mutations are highly associated with better responsive and survival outcomes of *PI3K* inhibitors in *HR+* breast cancer. The predictive and prognostic role of *PIK3CA* mutations will facilitate the diagnosis and prognosis of *HR+* breast cancer. More studies to explore the clinical superiority of *PI3K* inhibitors are warranted in *HR+/PIK3CA-*mutated breast cancer and other cancer forms.

## Figures and Tables

**Table 1:** Main characteristics of included studies.

Study and year	Study type	PI3K inhibitor regimen	Clinical outcome	NOS
Krop IE et al. 2016	Phase 2 trial. NCT01437566	Daily pictilisib 340 mg plus fulvestrant 500 mg	ORR and PFS	6
Vuylsteke P et al. 2016	Phase 2 PEGGY study. NCT01740336	Daily 260 mg pictilisib on day 1-5 each week, pulsed with paclitaxel	ORR and PFS	6
Loibl S et al. 2017	Phase 2 trial (NeoPHOEBE) NCT01816594	Neoadjuvant buparlisib plus trastuzumab and paclitaxel	ORR	6
Baselga J et al. 2017	Phase 3 BELLE-2; NCT01610284	Daily 100 mg buparlisib plus intramuscular fulvestrant 500 mg	ORR and PFS	8
Martin M et al. 2017	Phase 2/3 study. (BELLE-4) NCT01572727	Daily 100 mg buparlisib with paclitaxel	ORR and PFS	6
Di Leo A et al. 2018	Phase 3 BELLE-3 NCT01633060	Daily 100 mg buparlisib plus intramuscular fulvestrant 500 mg	PFS	7
Andre F et al. 2019	Phase 3 (Solar 1). NCT02437318	Daily 300 mg alpelisib plus 500 mg fulvestrant	ORR and PFS	8
Saura C et al. 2019	Phase 2 trial. NCT02273973	4 mg taselisib (5 days on, 2 days off) pulsed with daily 2.5 mg letrozole	ORR	6

## Data Availability

The data used and/or analyzed to support the findings in our study are available from the corresponding author upon reasonable request.
